# Tobacco use and oral sex practice among dental clinic attendees

**DOI:** 10.1371/journal.pone.0213729

**Published:** 2019-03-13

**Authors:** Neil H. Wood, Olalekan A. Ayo-Yusuf, Tshepo S. Gugushe, John-Paul Bogers

**Affiliations:** 1 Department of Periodontology and Oral Medicine, School of Oral Health Sciences, Sefako Makgatho Health Sciences University, Pretoria, South Africa; 2 Africa Centre for Tobacco Industry Monitoring & Policy Research, Sefako Makgatho Health Sciences University, Pretoria, South Africa; 3 School of Oral Health Sciences, Sefako Makgatho Health Sciences University, Pretoria, South Africa; 4 Faculty of Medicine and Health Sciences, Applied Molecular Biology Research Group (AMBIOR), University of Antwerp, Antwerp, Belgium; 5 Laboratory of Cell Biology & Histology, Antwerp, Belgium; University of Montana, UNITED STATES

## Abstract

Tobacco use and oral sex (OS) are important risk factors for oral and oropharyngeal Human papillomavirus (HPV) infection. Little is known about the prevalence of OS practice in South Africa. This study aimed to determine the prevalence of OS practice and tobacco use in a South African patient population. This cross-sectional study used a structured questionnaire to collect socio-demographic characteristics, tobacco use, betel nut use and OS practice data from consenting adults (≥18 years; n = 850). Oral sex practices were recorded for patients 18–45 years-old (n = 514). Data analysis included chi-square and multiple logistic regression analyses. Of the study population, 55.2% (n = 468) were female, 88% (n = 748) self-identified as black Africans and 45.1% (n = 383) were unemployed. Furthermore, 19.7% (n = 167), 6.4% (n = 54) and 2.1% (n = 18) were current smokers, snuff users and betel nut users, respectively. Out of the 514 who answered the questionnaire in relation to OS, 22.8% (n = 115) reported to practice it. Oral sex practice in the age group 18–45 years was most common among the self-identified white participants (41.9%); and among tobacco users than among non-tobacco users (30.9% vs. 20.5%; p = 0.022). A multivariable-adjusted regression model showed that white South Africans were more likely to use tobacco than black Africans (OR = 5.25; 95% CI = 2.21–12.47). The practice of OS was more likely among those 18–35 years-old (OR = 1.67; 95% CI = 1.01–2.74), but had no significant association with tobacco use (OR = 1.06; 95% CI = 0.62–1.83). The observed age and ethnic differences in both risk behaviours suggest a need for targeted population intervention in order to reduce the risk for oral HPV infection.

## Introduction

The aetiopathogenesis of oropharyngeal squamous cell carcinoma (SCC) has been linked to high-risk human papillomavirus (HPV) infection [1–3].While the incidence of SCC of the head and neck is diminishing, that of HPV-related oropharyngeal SCC is increasing [4]. This implies that different aetiologic mechanisms may be at play [5] and support the postulate that HPV-associated SCC is a distinct and separate clinical entity from tobacco and alcohol-associated SCC [6,7]. Earlier oral/oropharyngeal HPV studies were limited by the lack of a standardized meaning for the “oral” vs “oropharyngeal” anatomical compartments. This lead to ambiguity in some reports and care must be taken when interpreting results representative of these two distinct anatomic sites [8,9]. The oropharyngeal site is defined by Paquette and colleagues [9] as “…posterior one-third of the tongue, palatine and pharyngeal tonsils, bounded inferiorly by the epiglottis and superiorly by the soft palate.”.

Oral and oropharyngeal SCC is the 6^th^ most common cancer and also the 6^th^ largest cause of cancer related deaths worldwide [10]. Patients diagnosed with oral SCC have a mean 5-year survival rate of about 50%. The most important risk factors of oral SCC are tobacco smoking, excessive alcohol intake, chewing betel quid and areca nut and a diet low in fresh fruits and vegetables [10].

Tobacco use has a long association with the development of head and neck malignancy and the use of alcohol and tobacco are well-known risk factors for the development of head and neck SCC [3,11,12]. Some association between smoking and prevalence of oral HPV infection exists, but more importantly, tobacco use has been associated with a reduced capacity for the clearance of oncogenic HPV-infection [13,14]. Although the biologic link responsible for increased prevalence of oral HPV in current smokers has not yet been fully defined, the rationale lies in the local oral/oropharyngeal mucosal pro-inflammatory milieu and the immune suppression induced by tobacco use, creating a favourable niche for HPV infection and persistence [15].

Infection by HPV is the most common sexually transmitted disease (STD) [16]. Although oral and oropharyngeal HPV infections are believed to be acquired by orogenital contact with an infected sexual partner, by mouth-to-mouth contact or by autoinoculation from another infected site [17], some studies report the majority of cases with oral HPV infection are not the result of sexual transmission [18,19]. Nevertheless, it is important to understand the demographic characteristics of OS practice in order to further research on its influence in oral health, especially in resource-poor settings such as this study’s population.

HPV-infection and SCC of the mouth and oropharynx have been associated with patients becoming sexually active at a younger age, having numerous sexual partners, and with practicing orogenital sex (OS) [20–22]. While there is a strong association between HPV and oropharyngeal SCC with about 50% of all cases of HPV- cytopositive oropharyngeal SCC being caused by high-risk HPV genotypes, in the case of oral SCC there is limited evidence causally linking HPV infection of the mouth to oral SCC [23–25].

Within the limited scope of evidence, the apparently lower frequency of HPV infection in oral and oropharyngeal SCC of South African cohorts [8,26] could be because the practice of OS may be less common among South Africans than among Western and Asian populations; and may differ between different racial groups [27,28]. Reports on the ethnic distribution of OS practice are also very limited in the international literature, and when available, it presents different prevalence rates for OS practice according to the geographic region of the study [4,29,30]. While a number of studies have investigated the characteristics of tobacco use and to a lesser extent the practice oral sex [14], most have been done separately despite the fact that both risk behaviours may be related and co-exist. The practice of OS is a known high-risk sexual behaviour that facilitates oncogenic HPV transmission [31].

The purpose of this study was to investigate the prevalence of tobacco use and the practice of OS among the patients attending the Sefako Makgatho Health Sciences University Oral Health Centre located in a peri-urban area of South Africa.

## Material and methods

This cross-sectional study involved consenting adults (≥18 years; n = 847) who attended for consultation at a university-based Oral Health Centre (OHC). Using a structured self-administered questionnaire, socio-demographic characteristics that included age, gender, self-identified race/ethnicity (Black African; Coloured (Mixed ancestry); White; Indian/Asian), tobacco smoking and/or snuff use (some days or everyday), betel nut use and OS practices were recorded. Oral sex practices were recorded only for patients 18–45 years-old (n = 514). Oral sex practice was determined by asking participants whether they were currently engaged in oral sex practice, having their mouth in contact with a partner’s genitalia.

Data analysis included chi-square and multi-variable adjusted logistic regression analyses. Two separate regression models were reported for OS and tobacco use. In both instances the independent effect of one as a predictor-variable of the other as an outcome-variable was controlled for age, gender, ethnicity and employment status. All tests were two-tailed and p values of 0.05 or less considered as significant. Ethical clearance for this project was obtained from the Sefako Makgatho Health Sciences University Research Ethics Committee (MREC/D/187/2010:IR).

## Results

The study sample comprised 847 patients who visited the university-based Oral Health Centre. Study participants self-identified their race and of the 847 patients, 93% (748 patients) self-identified as black African, 6% (55 patients) were white, 2 patients (0.3%) were Indian, only 1 patient (0.1%) was of mixed race and 41 did not identify their race. Of the study population (n = 468), 55.2% were female and 26 participants did not indicate their gender on the questionnaire. Eighty-eight percent (n = 748) were Black and 45.1% (n = 383) were unemployed (Table 1). Owing to the small participation number, those participants who self-identified as ‘unknown race’, of Indian and of mixed race were excluded from further analysis.

**Table 1:** Socio-demographic characteristics of the study sample.

Characteristics		% (n)
Gender	Male	41.6 (n = 353)
	Female	55.2 (n = 468)
	Unknown	3.1 (n = 26)
Race	Black African	88.3 (n = 748)
	Whites	6.5 (n = 55)
	Indian/Asian	0.2 (n = 2)
	Mixed race	0.1 (n = 1)
	Unknown	4.8 (n = 41)
Age Group	18–35 years	44.2 (n = 368)
	36–45 years	22.5 (n = 187)
	>45 years	33.3 (n = 277)
Employment status	Employed	30.2 (n = 257)
	Retired/Student	7.6 (n = 64)
	Unemployed	45.1 (n = 383)
	Unknown	17.1 (n = 143)
Tobacco use	Current smoker	19.7 (n = 167)
	Current snuff user	6.4 (n = 54)
	Betel nut user	(n = 18)
Practice orogenital sex*	Current practice	22.8 (n = 115)

*Only among those 18–45 years old

Four hundred and seventy-five of the 748 black patients and 36 of the 55 white patients answered the question relating to their sexual behaviour; 21.6% (99) of the black patients and 41.9% (13) of the white patients practiced OS (Table 2). Data for other race groups were excluded from analysis due to insufficient sample size (n = 3).

**Table 2:** Association between socio-demographic characteristics, oro-genital sex and tobacco smoking.

Characteristics		Practice oro-genital sex* %	p-value	Tobacco use %	p-value
		(n = 514)		(n = 847)	
Gender	Male	32.1 (69)		38.6 (135)	
	Female	16.2 (46)		12.0 (56)	
			<0.001		<0.001
Race	Black African	21.6 (99)		21.7 (162)	
	Whites	41.9 (13)		53.7 (29)	
	Other' (Excluded)	0.5 (3)		0.0 (0)	
			0,031		<0.001
Age group	>45 years	n/a		25.8 (71)	
	36–45 years	17.4 (28)		23.0 (43)	
	18–35 years	25.2 (89)		21.5 (79)	
			0,05		0,432
Employment	Unemployed	24.8 (50)		23.8 (91)	
status	Retired/student/unknown	19.6 (31)		18.7 (39)	
	Employed	23.4 (36)		27.0 (69)	
			0,503		0,108
Tobacco user	No	20.5 (83)			
	Yes	30.9 (34)			
			0,022		
Practice oro-	No			19.1 (76)	
genital sex*	Yes			29.1 (34)	
					0,022

*Only among those 18–45 years that responded to this question

Of the participants, 19.7% (n = 167), 6.4% (n = 54) and 2.1% (n = 18) were current smokers, snuff users and betel nut users, respectively (Table 1). Out of the 514 who answered the questionnaire in relation to OS, 22.4% (n = 115) reported to practice OS (Table 2). Oral sex practice in the age group 18–45 years was significantly more common among white South Africans (41.9%) than among black South Africans; and among tobacco users than among non-tobacco users (30.9% vs. 20.5%; p = 0.022) (Table 2). A multivariable-adjusted regression model showed that compared to black South Africans, white South Africans were more likely to use tobacco (OR = 5.25; 95% CI = 2.21–12.47) and practice OS (OR = 2.38; 95% CI = 1.06–5.35). However, after controlling for confounding factors, the practice of OS was not significantly associated with tobacco use (OR = 1.06; 95% CI = 0.62–1.83) (Table 3).

**Table 3:** Multivariable-adjusted regression model of factors associated with tobacco use and practice of oro-genital sex among those 45 years old and younger.

Characteristics		Practice oro-genital sex* % (N = 514)	p-value	Tobacco use % (N = 847)	p-value
		OR (95% CI)		OR (95% CI)	
Gender	Male	1		1	
	Female	0.42 (0.27–0.67)	<0.001	0.15 (0.09–0.25)	<0.001
Race					
	Black African	1		1	
	Whites	2.38 (1.06–5.35)	0.035	5.25 (2.21–12.47)	<0.001
Age group					
	36–45 years	1		1	
	18–35 years	1.67 (1.01–2.74)	0.045	1.25 (0.74–2.13)	0.401
Employment status					
	Unemployed	1		1	
	Retired/Student/ unknown	1.15 (0.69–1.94)	0.593	0.50 (0.27–0.92)	0.026
	Employed	0.73 (0.41–1.31)	0.291	0.70 (0.40–1.23)	0.220
Tobacco user					
	No	1		-	
	Yes	1.08 (0.63–1.85)	0.776	-	
Practice oro-genital sex*	No	-		1	
	Yes	-		1.06 (0.62–1.83)	0.825

*Only among those 18–45 years that responded to this question

## Discussion

This study showed that about 1 in 5 clinic attendees in this dental training institution practice OS. Furthermore, almost a third of those who practice OS were also current tobacco users. Consistent with the literature, tobacco use were more common among men (38.6%) than among women (12%) and this ratio (3.2:1) is closer than that of the national prevalence (4:1) [32–33]. Our study indeed showed that tobacco use in this predominantly black African population of dental clinic attendees (21.7%) was slightly higher than the reported national prevalence of 17.7% for this population group [33,34].

Despite South African data showing that oropharyngeal cancer in white South African population occurs at a much older age than other ethnic groups [35], no reports on ethnic distribution of OS practice are available for the South African population. However, broader population based reports of OS practice demonstrate a wide variation between population groups.

Our finding of 32% prevalence of OS practice among males is comparable to 40% prevalence reported among high-risk male South African factory workers recently published [26]. However, the study by Vogt and colleagues [36] reports 84% of men and 82% of women in heterosexual couples practiced oral sex which was consistent with data from Canada (71%) [28] and the US (80%) [31]. Conversely, another South African study of heterosexual couples, but in a different geographic location, reported that only 8.7% of women and 6.2% of men reported to practice oral sex which is similar to that reported in China [37,38].

The differences in these reports could be due to different study designs, data collection methods, and analyses. The target population group also plays a role in the reporting of oral sex practice [28]. Conceivably, the practice of OS may be culturally inclined. The number of oral sex partners, the frequency of oral sexual events, and even the duration of each oral sexual event may all play a role in the extent to which OS practice is self-reported. However, these variables were not explored in detail due to the cultural and societal sensitivities surrounding this topic in this population group.

This study highlighted a significantly higher likelihood to practice OS among youth than older adults. This is consistent with the literature [28]. Furthermore, considering that OS is a significant source of exposure to HPV, OS may partly explain why HPV-associated oropharyngeal SCC is more common in younger people [10]. The practice of OS by younger adults has been characterised as a normative social practice that is less intimate and others do this in an effort to avoid pregnancy [39] and as a “benefit-provisioning mate retention behaviour” [40]. A study of 410 younger heterosexual adult women reported that OS was performed as a way to express love and care to their male partner [40]. The higher risk for OS among youths support targeted interventions such as the promotion of condom and dental dam in the prevention of oral HPV infection [41]

There were significant racial differences in the practice of OS and tobacco use with white South Africans most likely to report both risk behaviours for oral and oropharyngeal cancer. On the one hand, OS increases the risk of HPV-exposure and on the other hand, smoking reduces the clearance of HPV, which means that white South Africans who are more likely to both smoke and practice OS may be at a higher risk to develop oral and oropharyngeal infection. It is nevertheless pertinent to note that in this study, smoking was not significantly associated with OS practice, therefore neither of these risk behaviours can be used as a risk behaviour marker for the other.

The practice of OS was twice more common among white than black South Africans in this study. This relatively low frequency of OS, in particular among black South Africans, may explain why despite the fact that in South Africa the prevalence of genital HPV infection is as high as 22.1% among women [42] with one study demonstrating a prevalence of 68% [43], the prevalence of oral HPV infection (3.5–8.4%) [38,44] is relatively low. In fact, only about 20% of HIV-seropositive black women with genital HPV infection have concurrent oral HPV infection, and in only half of this 20% can the genital HPV genotypes be detected in the mouth [8]. Self-inoculation via the genital-oral route has been suggested as a source of oral HPV infection in the South African setting [38].

## Study limitations

Some caution in the interpretation of our study findings in relation to the study’s limitations would include the fact that the OS and tobacco behaviour were self-reported. It may indeed be that respondents provided sociably desirable responses and that this may be an under-representation of OS practice and of tobacco use. The findings of this study are limited to dental clinic attendees therefore may not be generalized to the general South African population.

Due to cultural and societal sensitivities associated with the practice of OS in this population group, the nature of the OS practice, including frequency of practice, was not further investigated. We believe that forcing this sensitive topic on this population would have greatly reduced participation and this project sensitised many participants and non-participants in this population to a topic considered taboo.

Despite these limitations, this study provides useful information for prioritizing public health interventions and for further research, which may include more in depth demographic and epidemiological profile of those who practice OS and the presentations of signs and symptoms of related infection.

## Conclusion

The study findings suggest that tobacco use and the practice of oral sex are not significantly associated risk behaviours and thus could be considered independent risks for oral and oropharyngeal infection. Furthermore, age and ethnic differences in both risk behaviours suggest need for targeted population intervention in order to prevent and reduce the incidence of oral and oropharyngeal infection. Community engagement and further investigation are required concerning perceptions of oral sex practice and tobacco use.

## Supporting information

S1 File Dataset submitted as per journal requirement. (XLSX)
